# The role of foot pressure measurement in the prediction and prevention of diabetic foot ulceration—A comprehensive review

**DOI:** 10.1002/dmrr.3258

**Published:** 2019-12-11

**Authors:** Katie E. Chatwin, Caroline A. Abbott, Andrew J.M. Boulton, Frank L. Bowling, Neil D. Reeves

**Affiliations:** ^1^ Musculoskeletal Science & Sports Medicine Research Centre, Department of Life Sciences, Faculty of Science & Engineering Manchester Metropolitan University Manchester UK; ^2^ Faculty of Biology, Medicine and Health University of Manchester Manchester UK; ^3^ Diabetes Research Institute University of Miami Miami Florida

**Keywords:** diabetic ulcer, peripheral neuropathy, plantar pressure, pressure feedback, pressure‐time integral

## Abstract

The predominant risk factor of diabetic foot ulcers (DFU), peripheral neuropathy, results in loss of protective sensation and is associated with abnormally high plantar pressures. DFU prevention strategies strive to reduce these high plantar pressures. Nevertheless, several constraints should be acknowledged regarding the research supporting the link between plantar pressure and DFUs, which may explain the low prediction ability reported in prospective studies. The majority of studies assess vertical, rather than shear, barefoot plantar pressure in laboratory‐based environments, rather than during daily activity. Few studies investigated previous DFU location‐specific pressure. Previous studies focus predominantly on walking, although studies monitoring activity suggest that more time is spent on other weight‐bearing activities, where a lower “peak” plantar pressure might be applied over a longer duration. Although further research is needed, this may indicate that an expression of cumulative pressure applied over time could be a more relevant parameter than peak pressure. Studies indicated that providing pressure feedback might reduce plantar pressures, with an emerging potential use of smart technology, however, further research is required. Further pressure analyses, across all weight‐bearing activities, referring to location‐specific pressures are required to improve our understanding of pressures resulting in DFUs and improve effectiveness of interventions.

## 1 INTRODUCTION

Currently 425 million adults have diabetes mellitus worldwide, however the prevalence is rising, with 629 million cases expected by 2045.^1^ Diabetes is the main cause of non‐traumatic lower limb amputations, of which up to 85% are the result of a diabetic foot ulcer (DFU).^2^, ^3^ Diabetic foot ulcers are a costly public health concern, with a large proportion leading to amputation or infection; DFUs are also associated with a reduced quality of life.^4^, ^5^ The lifetime risk of developing a DFU is 15‐25%.^6^, ^7^ However, once ulcerated, DFU recurrence rates are 40% within the first year and up to 65% after 5 years post‐healing.^8^, ^9^ Risk factors for DFU include diabetic peripheral neuropathy, foot deformity and trauma, with diabetic peripheral neuropathy being the predominant risk factor.^5^, ^10^, ^11^, ^12^

The purpose of this review is to explore the role of high plantar pressure, which accumulates due to a number of risk factors, in the prediction and prevention of DFU. The authors review the different methods of plantar pressure assessment in both barefoot and in‐shoe conditions, as well as the pressure parameters analysed in previous literature. Studies assessing plantar pressure typically find pressure to be higher for people with diabetes and higher still for ulcerated cohorts. However, despite this, vertical plantar pressure alone is still reported as a poor predictor of DFU in prospective studies. The review discusses the relative merits and limitations of previous studies, which may have contributed to low predictive ability and the extent to which previous methods may relate to pressures experienced throughout “real‐life” daily activity.

### 1.1 Factors resulting in high plantar pressure

Diabetic peripheral neuropathy leads to a loss of protective sensation resulting in abnormally high, repetitive and undetected pressures applied to the weight‐bearing plantar surface of the foot. In addition, foot deformities such as hammertoe and small muscle wasting further contribute to increased plantar pressure, particularly at the metatarsal heads where bony prominences reside.^13^ Other factors including a reduced ankle dorsiflexion and reduced plantar tissue thickness are also reported to contribute towards increasing plantar pressure.^10^, ^14^ High plantar pressures lead to thickening of callus, putting added pressure on the underlying soft tissue and leading to tissue breakdown and ulceration.^15^, ^16^

Current DFU prevention interventions focus on reducing these high plantar pressures.^17^ In the high‐risk diabetic foot, custom‐made footwear and/or insoles are often prescribed which aim to offload pressure from high‐risk areas by accommodating foot deformities. When worn, these interventions have been shown to significantly reduce ulceration rates.^18^, ^19^ However, footwear interventions are often associated with poor adherence, thus limiting their effectiveness.^20^, ^21^, ^22^ Although the aim of prescription footwear is to reduce plantar pressure, the previous supporting research on the link between high plantar pressure and DFU risk is associated with some limitations, as discussed in the sections below.

## 2 BAREFOOT PRESSURE ANALYSIS

**Figure 1:**
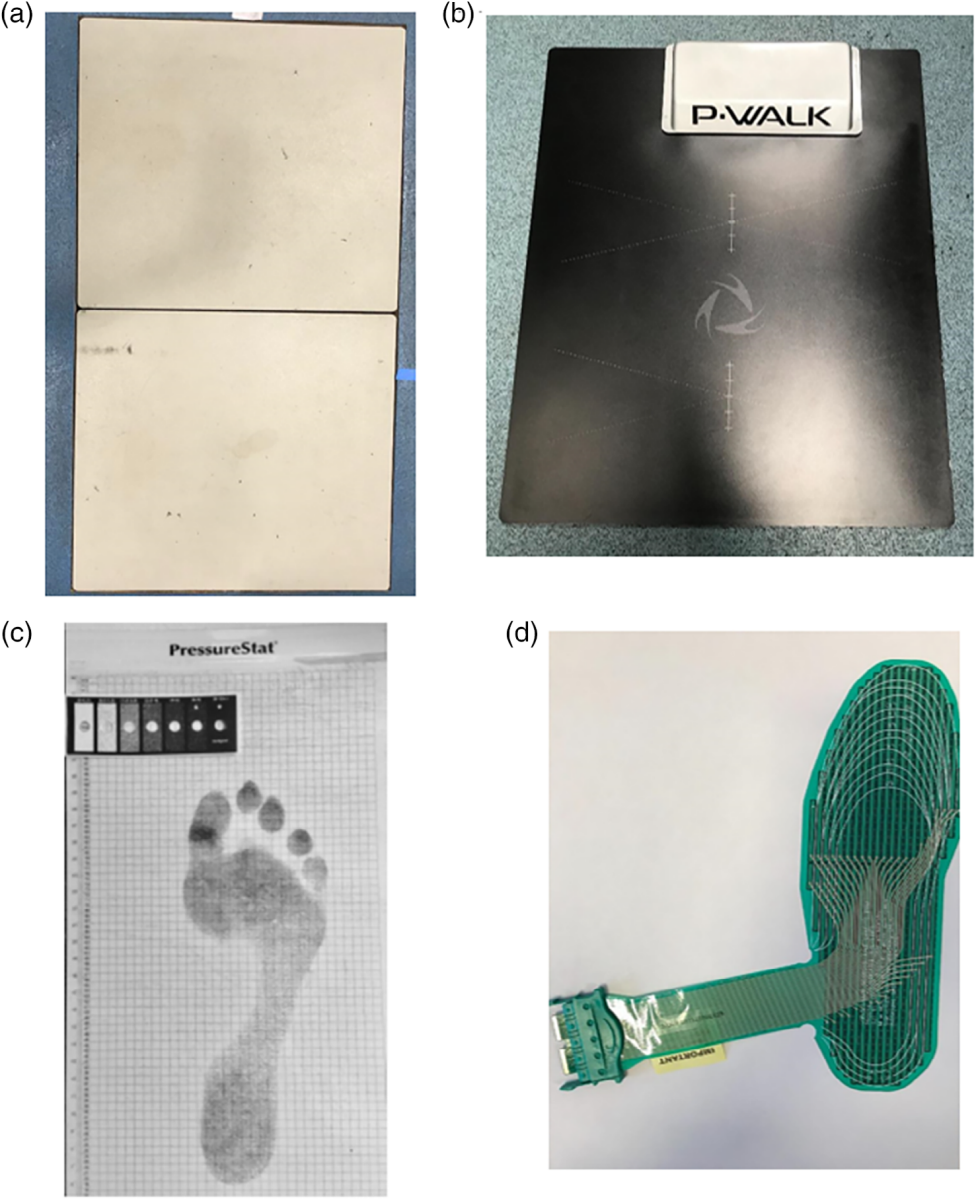
Examples of equipment used to measure plantar pressure. A, AMTI force platform (Advanced Medical Technology, Inc. Watertown, MA). B, BTS P‐walk pressure plate (MA). C, PressureStat (Medical Gait Technology BY, Emmen, The Netherlands). D, F‐scan pressure assessment system insole (Tekscan, Inc., Boston, MA). The equipment A‐C are typically used to collect barefoot pressure data, whereas D is placed in‐shoe

Many studies investigating plantar pressure within the diabetic cohort have done so using barefoot pressure analysis, predominantly using pressure platforms (Figure 1).^23^, ^24^, ^25^, ^26^, ^27^ Such measurements take place inside a laboratory and involve the participant walking along a walkway ensuring successful foot placement within the platform. However, methodology and patient characteristics vary within the literature (Table. 1). Vertical plantar pressure is primarily assessed, however studies either focus on the foot as a whole, or investigate pressure at specific plantar locations, with the majority focusing on the forefoot. Only a minority of studies analyse pressure specific to ulcer location. Although some variability exists, the consensus from the literature is that diabetes patients, particularly those with a history of DFU, have higher plantar pressures than controls.^12^, ^35^, ^38^

**Table 1:** Demographic data for participants classified into selected groups, in reviewed plantar pressure studies

First Author (Year)	Ulcerated group (DU)							No in‐study ulcer, neuropathic group (DPN)							Diabetes control group (DC)					Healthy controls (HC)			
	DFU (n=)	DFU History (n=)	% Type 2	% Male	Age (years)	BMI (kg/m^2^)	Diabetes duration (years)	DPN (n=)	DFU History (n=)	% Type 2	% Male	Age (years)	BMI (kg/m^2^)	Diabetes duration (years)	DC (n=)	% Type 2	% Male	Age (years)	BMI (kg/m^2^)	HC (n=)	% Male	Age (years)	BMI (kg/m^2^)
Abbott (2017)^28^	9	9	—	88.8	62.6 (11.5)	31.2 (8.2)	—	6	6	—	100	56.7 (7.1)	32.3 (6.2)	—	*no DC group*					12	33.3	58.0 (8.3)	26.2 (3.8)
Armstrong (1998)^29^	70	—	—	74	52.3 (10.3)	30.9 (5.7)	14.3 (9.2)	149	0	—	33	51.8 (10.4)	32.3 (6.2)	9.2 (8.8)	*no DC group*					*no HC group*			
Bacarin (2009)^30^	10	10	90	80	58.2 (6.7)	27 (5.5)	17.5 (9.3)	17	0	94	47	54.7 (7.8)	26.1 (4.6)	13.4 (8.4)	*no DC group*					20	35	48.7 (9.4)	24.3 (2.6)
aFrykberg (1998)^31^	99	99	70.7	69.7	60 (10.5)	29.4 (5.5)	17 (9.5)	152	0	86.8	62.5	57 (13.5)	30.5 (6.8)	12 (10.8)	*no DC group*					*no HC group*			
bLedoux (2013)^32^	47			95.7	68	30	19	544	—	—	98.3	67	30.3	15	*no DC group*					*no HC group*			
cMelai. (2011) 33	*no ulcer group*							76	—	100	—	66 (7.2)	31.18	—	33	100	—	62.8 (7.1)	31.0	19	—	68.1 (5.2)	24.3
dOwings (2009)^34^	*no ulcer group*							49	49	—	77.6	62.9 (10.3)	28.1	—	*no DC group*					*no HC group*			
aPham (2000)^35^	73	32	76.7	67.1	59 (11)	29.6 (7.1)	16 (12)	175	55	81.7	42.9	58 (13)	31.3 (7.0)	13 (10)	*no DC group*					*no HC group*			
cSacco (2009)^36^	*no ulcer group*							15	—	100	60	57 (6)	28.2	>5	*no DC group*					16	31	46 (11)	25.3
e ^,^ fStess (1997)^37^	49	49	—	—	61.7 (12.4)	30	—	14	0	—	—	66.0 (8.9)	30.6	—	34	—	—	66.6 (9.1)	28.6	*no HC group*			
Waaijman (2014)^12^	71	71	71.8	85.9	62.8 (11.2)	30.6 (6.2)	16.7 (13.2)	100	100	71	80	63.6 (9.4)	30.7 (5.3)	17.7 (13.8)	*no DC group*					*no HC group*			

*Note*: Reported means and standard deviations (SD) when available. Diabetes duration was omitted from the DC group as it was not provided in any of the studies, as was ulcer history. Diabetes control = no neuropathy and no ulcer.

a Within the DPN group, not all participants are thought to have peripheral neuropathy based on reported mean (SD) VPT scores, exact numbers of neuropathy patents were not provided.

b Not all participants within this study had peripheral neuropathy, however the majority did: DU ‐ 38/47, DPN ‐ 259/544.

c These studies did not mention previous ulcer history, however active ulcers were excluded.

d This study included only one group of participants who had remained healed following previous ulceration.

e Study states predominantly males but does not give percentage.

f Mean (SE) values were reported in this study.

### 2.1 Whole‐foot barefoot analysis

A number of previous studies conducting barefoot pressure analysis have calculated peak plantar pressure of the whole‐foot, rather than specifying location. Such studies vary in methodology, with some averaging peak plantar pressure from mid‐gait steps with the platform placed along a walkway,^31^, ^35^ whereas other studies implement a two‐step approach to the platform.^12^, ^25^ Research suggests the two‐step approach not only reduces time spent barefoot walking and the associated risk to insensate feet, but also reduces the difficulty of making full contact within the boundaries of the platform.^39^, ^40^ However, familiarisation and repetition of walking trials are still required to ensure as natural gait as possible, thus still imposing some element of potential risk on the high‐risk diabetic foot as part of the barefoot testing procedure.

Prospective studies consistently report significantly greater baseline peak plantar pressure in diabetes participants who ulcerated within the follow‐up period, compared to those that remained ulcer‐free (Table. 2).^12^, ^25^, ^35^ However, the majority of these studies included patients with and without a history of DFU. Individuals with a history of DFU are reported to have significantly higher plantar pressures than those without DFU history; therefore including participants without DFU history in such studies may have diluted the results and contributed to the low sensitivity of pressure predicting ulceration.^30^ Grouping together participants with active and previously healed DFUs, as demonstrated in a previous cross‐sectional study by Frykberg et al.^31^ may weaken conclusions drawn about the causal relationship between high plantar pressure and DFU, due to patients with active DFUs potentially altering their gait (albeit without any sensory feedback) to avoid any further damage to the active wound.^27^ Alterations in gait, and consequently plantar pressures, are expected to differ depending on DFU status; therefore, analysis should ideally group patients accordingly.^41^ Frykberg et al.^31^ also found significantly greater peak plantar pressure for the ulcerated cohort compared to the non‐ulcerated cohort. In contrast to many whole‐foot barefoot studies, Lavery et al.^25^ described recording the location of the peak pressure, however, as is the case with most whole‐foot barefoot studies, did not report the location nor conduct any location‐specific pressure analysis. More comprehensive pressure analyses, which take into account any effects of location on pressure and DFU, as well as more stringent patient grouping, may improve DFU prediction.

**Table 2:** Barefoot and in‐shoe plantar pressure data of selected participant groups, taking into account plantar area of pressure measurement, between studies

First Author (Year)			Peak Plantar Pressure (kPa)																			
	Foot pressure measurement		Whole foot				Forefoot				Midfoot				Rearfoot				Hallux			
	System	Specifications	DU	DPN	DC	HC	DU	DPN	DC	HC	DU	DPN	DC	HC	DU	DPN	DC	HC	DU	DPN	DC	HC
*Barefoot studies*																						
Abbott (2017)^28^	PressureStat																		449 (178)	231 (107)		237 (61.8)
Armstrong (1998)^29^	EMED	4pixels per cm^2^					831 (247)	627 (214)														
a ^,^ bBacarin (2009)^30^	Pedar	50 Hz, 1.6‐2.2cm^2^					367 (86.2)	368 (89.2)		348 (88.4)	291 (152)	205 (119)		139 (76.4)	342 (119)	342 (76.9)		337 (95.9)	270 (137)	306 (112)		307 (111)
Frykberg (1998)^31^	F‐Scan	5mm^2^	657 (304)	481 (235)																		
Lavery (2003)^25^	EMED	4pixels/cm^2^	955 (264)	851 (273)																		
c ^,^ dMelai (2011)^33^	EMED	100 Hz, 4 sensors/cm^2^						501 (198)	448 (133)	364 (75)		150 (52)	165 (60)	118 (24)		425 (118)	419 (109)	359 (93)		463 (243)	514 (286)	355 (149)
Owings (2009)^34^	EMED	50mm^2^						566 (316)												486 (242)		
Pham (2000)^35^	F‐Scan		706 (373)	522 (255)																		
a ^,^ eSacco (2009)^36^	Pedar	100 Hz						246 (56.3)		219 (35.3)		114 (52.2)		75.7 (31.1)		220 (40.4)		197 (27.8)				
fStess (1997)^37^	EMED						480 (18)	405 (28)	407 (17)													
Waaijman (2014)^12^	EMED	50 Hz, 4 sensors/cm^2^	1042 (260)	935 (307)																		
*In‐shoe studies*																						
gLedoux (2013)^32^	F‐Scan		219 (16)	194 (2)			383 (50)	303 (5)			267 (85)	141 (2)			241 (27)	266 (3)			172 (20)	200 (4)		
Owings (2009)^34^	Pedar	1.85 cm^2^						207 (68)												214 (71)		
	Pliance	0.194 cm^2^						291 (132)												304 (124)		
Waaijman (2014)^12^	Pedar	50 Hz, 1 cm^2^	261 (83)	249 (77)																		

*Note*: Reported mean (SD) peak plantar pressure (kPa) while walking.

Abbreviations: DC, diabetes control group with no DFU history and no peripheral neuropathy; DPN, diabetes patients with peripheral neuropathy who did not ulcerate in‐study; DU, diabetes patients who developed an ulcer in‐study; HC, non‐diabetic, healthy controls.

a These studies placed in‐soles in socks to record pressure.

b This study split forefoot into medial and lateral, the highest values were reported, lateral for DU and DPN, medial for HC.

c This study split forefoot into the five metatarsal heads, the highest value (third MTH) are shown.

d Some analysis was conducted using a sensor specification of 50 Hz, 2 sensors/cm^2^.

e Reported pressure at heel strike and push‐off, used value from heel strike for rearfoot and push‐off for forefoot and midfoot as these were the highest.

f Mean (SE) values were reported in this study.

g This study split the forefoot into multiple sites, the location with the highest value was used: DU – first MTH, DPN – second‐fourth MTH.

Another suggested explanation for vertical plantar pressure being a poor predictor of DFU, is not taking shear plantar pressure into consideration.^42^, ^43^ The majority of studies focus on vertical plantar pressure rather than shear, potentially due to its greater magnitude and ease of measurement with commercial systems compared to shear pressure.^44^ However, investigating shear pressure may increase the understanding of plantar foot mechanics and their role in the development of DFU.^45^ The few studies that did measure both parameters, found no general trend in the locations of the peak shear and vertical plantar, with the majority of participants having peak shear and peak vertical pressure occurring at different sites.^42^, ^43^, ^45^ Furthermore, even fewer papers related peak shear pressure to DFU. Yavuz et al.^43^ found more sites of peak shear to match sites of recently healed forefoot DFUs compared to peak vertical only sites, however, such differences were small. In addition, DFUs also occurred at sites where both peak shear and peak vertical plantar pressures were at the same location, as well as sites of neither peak parameters. Such results perhaps highlight the complex, multifactorial nature of DFU. Similarly Yavuz et al.^46^ also investigated shear in relation to DFU, however on this occasion compared the magnitudes of peak shear and vertical plantar pressure between diabetes participants with and without a history of DFU, which authors believed to be the first of its kind. Both peak shear and vertical plantar pressures were higher in the DFU group, but only shear reached significance. However, the authors did suggest their study might have been underpowered to detect a significant difference in peak vertical pressure, but believed the result to be clinically meaningful. The above studies measured shear pressure while barefoot and so results are unlikely to represent shear pressure applied in‐shoe, which may also differ depending on footwear.^45^ Therefore, further investigation into in‐shoe shear pressure with larger cohorts and of a longitudinal design are required before we can fully understand the role of shear pressure in the development of DFU. However currently, only a limited number of commercial devices are available that are capable of measuring in‐shoe shear pressure. Nevertheless, existing research does suggest measuring both shear and vertical plantar pressure along with other risk factors could be beneficial in improving the understanding and prediction of DFU. Although as suggested throughout this review, more ecologically valid research (ie, research that translates well to real‐life settings) is needed before ruling out plantar pressure as a sole predictor of DFU.

### 2.2 Location‐specific barefoot pressure analysis

To provide more detail, studies have identified peak vertical plantar pressures that are region specific. Such research often reports high ulceration rates at the forefoot, for example, Caselli et al.^24^ reported 98% of DFUs within a 30‐month follow‐up to be located at the forefoot. Therefore, the forefoot has been a particular focus of interest for measuring region‐specific pressures.

Certain cross‐sectional studies have focused on barefoot forefoot pressures alone, results of which follow a similar pattern to that of whole‐foot analysis, with the ulcerated cohort displaying significantly higher peak plantar pressure.^29^, ^37^ However, similar to Frykberg et al.,^31^ studies included active and healed DFUs within their “ulcerated” cohorts, which may have contributed to forefoot pressure alone not being able to identify accurately patients at risk of ulceration.^29^ On the other hand, following a 30‐month prospective study Caselli et al.^24^ reported that forefoot peak pressure was able to accurately predict ulceration, as was the ratio of forefoot to rearfoot pressure. However, patients were grouped by severity of neuropathy, without reference to their DFU history. Forefoot and rearfoot pressure were both significantly higher for moderate to severe cases of neuropathy, which are predominantly at high risk of ulceration.^12^ In addition, the forefoot to rearfoot ratio highlighted an imbalance in pressure distribution, particularly for those with severe neuropathy. Such findings highlight the need for location specific pressure analysis rather than analysing the foot as a whole.

A small number of studies have provided further detail by separating barefoot pressure into more regions. Sacco et al.^36^ sectioned the foot into rearfoot, midfoot and forefoot, whereas Bacarin et al.^30^ looked at five regions, by splitting the forefoot into medial, lateral and the hallux. While still assessing barefoot pressure, these studies adopted an alternative method by using insoles placed in socks, which participants wore while walking without shoes. Such approach allowed for multiple steps per trial, without the possibility of altering gait to ensure contact with any platform.^44^, ^47^ Sacco et al.^36^ compared non‐diabetic individuals to patients with diabetic neuropathy; however, DFU history was not reported. Bacarin et al.^30^ went further and included three patient groups: non‐diabetic, diabetic neuropathy with and without history of DFU. Although the diabetic cohorts showed greater peak pressures at all regions, Sacco et al.^36^ found only the midfoot and forefoot during push‐off to be significantly greater, whereas Bacarin et al.^30^ found the group with a history of DFU to have significantly higher pressure at the midfoot region only, compared to no DFU history and non‐diabetic participant groups. Other regions showed little difference between diabetes groups. Pressure at the rearfoot also showed similar values to non‐diabetic controls. Such results provide more detail than previously described whole‐foot studies and did not as perhaps expected, indicate that pressure may differ depending on location. More research is needed to confirm such results.

### 2.3 Barefoot pressure analysis specific to ulcer location

To the authors' knowledge, there have only been two studies assessing barefoot pressure at the site of previous ulceration. Although different in study design, results suggest the location of ulceration relates to the magnitude of pressure at that particular site.^12^, ^28^ A prospective study assessed barefoot plantar pressure using a pressure platform at the site of previous ulceration, using similar methods to previously discussed barefoot studies. Patients who re‐ulcerated at the same site within the follow‐up period had significantly higher pressure than patients who did not re‐ulcerate at that specific site, or ulcerated elsewhere.^12^ While this study provides an interesting insight into location specific pressure and re‐ulceration, information on any specific location on the plantar foot or comparison to a control group is missing. A recent cross‐sectional study considered such limitations and identified a site‐specific relationship at the hallux.^28^ Barefoot pressure at the hallux, which was measured using the PressureStat footprint map, was greater for diabetes patients with a previous hallux DFU, compared to a group of diabetes patients with a history of ulceration at another site and compared to a group of non‐diabetic controls. The PressureStat, a semi‐quantitative footprint map, is an easy and inexpensive method of highlighting any specific regions of high plantar pressure, which are determined by comparing the greyscale of the footprint to a calibration card.^48^ However, analysis using a visual scale can be subjective, combined with general limitations of barefoot analysis. Therefore, further investigation using less subjective analysis is required to confirm site‐specific relationships between plantar pressure and DFUs.

Separating plantar pressure analysis into regions may provide more detail, however barefoot analysis may be open to criticism because patients with diabetic neuropathy are advised against walking barefoot, due to the risks of injury; furthermore, barefoot pressure analysis may not be indicative of pressures experienced on a daily basis, which ultimately lead to ulceration. Nevertheless, barefoot analysis does provide a “fundamental” measure of plantar pressures without the potentially confounding/pressure‐modifying effects of footwear and/or orthotics and so for certain purposes may be informative.

Most daily activity takes place while wearing shoes for patients with diabetic neuropathy. Gait biomechanics, including plantar pressure, differ between barefoot and shod conditions. Therefore, some studies suggest that a more ecologically valid approach of analysing daily life plantar pressure is to do so in shod conditions.^34^

## 3 IN‐SHOE PRESSURE ANALYSIS

Individuals with diabetic neuropathy are advised to always wear footwear during daily activities in order to reduce pressure and chance of trauma to the foot.^12^, ^34^, ^49^ Studies where both in‐shoe and barefoot pressure are assessed support such guidelines by consistently reporting plantar pressures to be lower in‐shoe.^12^, ^34^ However, patients following these guidelines still ulcerate and so the analysis of in‐shoe pressure is an important feature within the literature.

An example of an in‐shoe vertical pressure sensor is shown in Figure 1. However, developing sensors to measure in‐shoe shear pressure has proved to be more of a challenge.^50^ Although there have been advancements in the measurement of in‐shoe shear pressure, studies investigating in‐shoe shear in relation to DFU are near non‐existent.^51^

### 3.1 In‐shoe pressure analysis in relation to DFU risk

Studies generally show that vertical plantar pressures experienced in‐shoe are lower than barefoot analysis, however those who ulcerate still have greater in‐shoe vertical pressures than cohorts who remain ulcer‐free. Advantages and disadvantages of barefoot and in‐shoe pressure analysis are highlighted in Table 3. A threshold of 200 kPa for vertical plantar pressure has been suggested within in‐shoe pressure research, to highlight those at risk of DFU.^34^ While the majority of the cohort's average pressure data remains in line with this threshold, some individuals who remained ulcer‐free did have pressure above the threshold and some who ulcerated had pressures below this threshold. Furthermore, one study reported 36% of ulcer‐free patients and 51% of patients who ulcerated to have pressures above the threshold.^12^

**Table 3:** Advantages and disadvantages of barefoot and in‐shoe pressure assessment methods

Assessment type	Advantages	Disadvantages
Barefoot	Easy to use Durable Embedded in floor to allow normal gait Allows assessment of “base” plantar pressure development without footwear	Restricted to laboratories Requires familiarisation to ensure natural gait Can be limited by patient's ability to make contact with the platform Requires multiple trials Walking barefoot presents a risk to diabetic neuropathy patients Does not account for pressure‐reducing nature of footwear
In‐shoe	Portable system Allows multiple footsteps per trial Less risk to the diabetic foot Allows assessment of pressure‐reducing nature of footwear	Majority of systems involve the participant being tethered by cables Possibility of sensor slipping and becoming damaged

Studies assessing in‐shoe pressure tend to be more location‐specific. A few studies focused on in‐shoe pressure analysis at the site of a previous DFU, once again showing similar results to barefoot analysis, however further research is required.^12^, ^32^, ^34^ To the authors' knowledge, only one study separated pressure analysis at previous DFU sites into regions, instead of combining all DFU data.^32^ Although the study conducted no statistical analysis to compare pressure data, the combined pressure at sites of ulceration was higher than pressure at the same site in non‐ulcerated patients. However, when looking at location‐specific data, the hallux and heel, which had the highest DFU rates along with the metatarsals, had lower peak plantar pressure than the non‐ulcerated cohort, whereas peak plantar pressure was greater for the ulcerated metatarsals, compared to non‐ulcerated. Furthermore, higher baseline peak plantar pressure was only significantly associated with an increased DFU risk at the metatarsals, potentially indicating a location‐specific relationship at the metatarsals only. However, although including a large sample size, only five mid‐gait steps per foot were analysed, whereas Arts and Bus^52^ suggest twelve steps are required to ensure reliable and valid in‐shoe pressure data. In addition, 50% of the whole cohort and 19% of ulcerated cohort were non‐neuropathic, yet neuropathy is a central risk factor for DFU. Including non‐neuropathic patients gives reason to expect some DFUs were not neuropathic plantar ulcers and may have developed through a different pathway, unrelated to plantar pressure, potentially complicating the results. Therefore, further analysis is required to confirm whether a location‐specific pressure and ulceration relationship exists for neuropathic DFUs.

### 3.2 Is in‐shoe pressure indicative of pressures experienced in day‐to‐day life?

In‐shoe pressure analysis removes the need for directed walking over a pressure platform and allows the analysis of consecutive steps. Although more indicative of pressures experienced by an individual with diabetic peripheral neuropathy during daily‐life, through incorporating footwear and insoles, the majority of studies have still only assessed a “snapshot” of in‐shoe pressure during one laboratory visit. However, one prospective study did assess in‐shoe pressure at follow‐up visits, results of which were averaged over two consecutive visits to indicate loading over the three months in between.^12^ While such methods may be more representative than a single measurement of in‐shoe pressure, assumptions concerning the loading between the 3‐month study visits may not be evidence‐based. Furthermore, in‐shoe pressure data collection involves participants being tethered to cables, limiting the extent of movement. In addition, as with the majority of barefoot and in‐shoe studies, pressure was assessed during level, straight‐line walking only and thus may still not be representative of habitual gait during all daily activities. Nevertheless, a small number of studies have assessed pressure during additional walking activities including walking in a circle, ascending and descending a ramp and staircase.^53^, ^54^ However, one study included patients with low levels of foot deformity, no history of foot trauma and no description of any DFU history, thus indicating patients likely had little risk of plantar ulceration and the associated higher plantar pressures. Such patient demographics perhaps contributed to the surprisingly significantly greater pressures in all activities for the non‐neuropathic participants.^54^ A second study did include higher risk patients, 44% of whom had a history of DFU, however, no within‐patient comparisons took place and instead the comparably small sample size formed a single cohort, to compare pressures between different walking conditions.^53^ Both studies found level walking to produce the highest pressures for the most part, but suggested such results may be due to patients walking slower in other tasks compared to level walking. Furthermore, ecological validity is somewhat questioned for both studies due to patients wearing standardised shoes, when in fact the majority of the neuropathic diabetes population wear custom‐made shoes.^20^, ^55^ Further research with larger cohorts of at‐risk patients completing different activities is required to confirm such results and improve our knowledge of pressures experienced on a daily basis.

## 4 INFLUENCE OF DAILY ACTIVITY ON DFU DEVELOPMENT

Research suggests the formula for the development of a DFU includes the product of plantar pressure and repetitive loading. The amount of activity an individual undertakes is often used to help estimate the cumulative pressure exerted on the plantar foot. It has been proposed that the more active a person with diabetic neuropathy is, the greater the cumulative pressure exerted and the greater the risk of ulceration.^56^ As discussed previously, pressure analysis of the diabetes population has focused on walking; this is also the case for the majority of studies assessing activity. Studies often record the number of steps per day as an indication of weight‐bearing physical activity.^57^, ^58^ However, although increased cumulative loading is thought to lead to a DFU, studies have shown that patients with a history of DFU walk significantly fewer steps per day than people with no history of DFU and healthy controls.^56^, ^57^, ^58^ An accelerometer is regularly the device of choice for measuring activity, however, such data is usually collected over a short period of time (eg, 1 week) and so may not adequately capture activity levels of diabetes patients, particularly those who are at risk of DFU, which are reported to be variable.^56^ Alternatively, Lemaster et al.^59^ used questionnaires to record self‐reported activity of the previous 24 hours, every 17 weeks for two years. Unlike previously mentioned studies, this study included all weight‐bearing activities, including standing and sitting, which are likely to contribute to the cumulative pressure exerted on the plantar foot and associated DFU risk. However, there was limited analysis on the different types of activity, apart from at baseline, where patients with a prior DFU spent more hours sitting than walking. Furthermore, Lemaster et al.^59^ reported no significant differences in weight‐bearing activity between participants who ulcerated within the follow‐up and those who did not, in fact, higher activity levels were reported to reduce the risk of ulceration, which conflicts previous theories. In addition, participants with neuropathy were slightly less active than those with intact sensation; however, such differences were not significant. Although activities other than walking were considered, activity over the prior 24 hours was assumed to remain constant throughout each 17 week time period between questionnaires. In addition, the questionnaire was reported to have strong validity with a step‐activity monitor; however, in terms of distinguishing between different types of weight‐bearing activity, the sensitivity of this measure may be questionable.

A more sensitive method of distinguishing between activity types than a questionnaire, is a triaxial accelerometer, as reported by Najafi et al^60^ Participants, all of whom had peripheral neuropathy, spent more time sitting and standing compared to walking, a similar finding to that suggested by Lemaster et al.^59^ at baseline. However, results were not compared to a control group and analysis took place over 48 hours only. Furthermore, there was no mention of any foot deformities or previous DFUs, indicating that participants may have been lower risk than previously studied cohorts and this was also indicated by a higher step‐count. Nevertheless, such results are promising and highlight the importance of future studies measuring all types of weight‐bearing activity, as ultimately all contribute to the pressure and cumulative loading applied to the plantar foot and associated DFU risk. Future studies should compare the activity of high‐risk patients to controls, with accelerometers worn for a longer duration.

## 5 RELEVANCE OF CUMULATIVE PRESSURE DATA FOR DFU RISK

Although further research is needed, previous studies suggest that diabetic patients at risk of ulceration spend more time standing and sitting, than walking.^59^, ^60^ Individuals are still at risk of ulcerating during such weight‐bearing activities, yet pressure assessment of the diabetes population has been limited to walking only.^53^, ^54^ Compared to walking, other weight‐bearing activities such as standing typically have lower peak pressures; however, this pressure is applied for longer. Prolonged pressure increases the duration of blood occlusion and the associated plantar tissue ischaemia, increasing the risk of developing a DFU.^61^ Therefore, a cumulative measure of pressure applied over a given time such as pressure‐time integral data, which takes into account loading time, may be more indicative of DFU risk than peak pressure; however, such analysis only exists for walking.^33^, ^62^, ^63^

Pressure‐time integral data is occasionally reported alongside the parameter of choice, peak pressure, with conflicting views as to whether it adds any benefit.^63^ The majority of studies reporting both parameters found no differences between them, essentially, any significant result or pattern reported for peak pressure was also present for the pressure‐time integral.^52^, ^54^, ^64^ The few studies that did find differences, perhaps indicating a benefit of reporting both, were associated with some limitations. Differences were only evident at the heel, likely due to its greater variability during stance compared to other areas.^30^, ^65^ The heel is not a typical region of ulceration and so such result has limited clinical relevance. Furthermore, other studies that found a difference between parameters did not standardise walking speed.^53^, ^66^ Walking speed affects pressure‐time integral more than peak pressure and, if standardised, differences would be expected to be minimal. In addition, pressure‐time integral data combined with strides per day was used to estimate cumulative plantar pressure.^58^ While this may provide a more accurate estimation of cumulative pressure compared to using either measurement alone, again, the only activity assessed was walking. Further investigation into pressure parameters of all weight‐bearing activities of daily‐life is required. Peak, pressure‐time integral and cumulative pressure data may best suit different weight‐bearing activities, however, conclusions cannot be made until such analysis has taken place within the diabetes cohort.

## 6 PLANTAR OFFLOADING INTERVENTIONS FOR THE AT‐RISK FOOT

In clinical practice, offloading interventions such as footwear and insoles are commonly prescribed to reduce high plantar pressure in an attempt to heal or prevent DFUs. The main purpose of such interventions are to reduce plantar pressure to an active DFU or areas at‐risk of developing a DFU by transferring pressure to other foot regions or to the offloading device.^67^, ^68^, ^69^

As discussed in previous sections, plantar pressure is lower in‐shoe than in barefoot conditions, therefore in an attempt to prevent ulceration, custom‐made therapeutic footwear are commonly prescribed to offload the foot regions of interest; however, ulcerations still may occur while wearing such footwear.^70^ Although offloading high plantar pressures is the main aim of footwear prescription, the measurement of plantar pressure does often not play a role in footwear design and manufacturing.^71^, ^72^ Instead, clinical judgement and foot shape are taken into account, which vary in method, in addition to a wide variety of materials being used.^73^ Therefore, due to large variability within both research and clinical practice, there are no standardised protocols and so footwear development is often described as more of an art than a science.^71^, ^74^, ^75^

Of the many footwear designs available, those with a rocker‐bottom outsole, designed to compensate for minimal movement at the joints of the foot and ankle, as well as maximise foot contact area, have consistently been shown to reduce forefoot pressure, whereas other designs have shown variable results.^76^, ^77^, ^78^ To further facilitate plantar offloading, the inclusion of an insole is a vital component of therapeutic footwear and has been shown to significantly reduce plantar pressure compared to footwear alone.^79^, ^80^ To ensure successful offloading a custom‐made insole is desirable over off‐the‐shelf alternatives.^71^, ^74^ Insoles are often customised using an impression of foot shape and clinical judgement; however, the addition of barefoot pressure assessment to this design process has seen significant improvements to offloading capabilities along with a reduction in DFU recurrence.^74^, ^79^, ^81^ Barefoot pressure analysis was used to identify areas of high pressure to guide the insole design process and while for the most part this was successful, there was evidence of some variability between individuals, with some seeing no benefit of the additional barefoot pressure input. The use of barefoot pressure to guide off‐loading taking place in‐shoe perhaps might contribute to some of this variability, as footwear could alter the plantar pressure profile. Studies that modified insoles based on in‐shoe pressure also reported significant reductions in plantar pressure following modifications.^49^, ^70^, ^72^ However, one study found no significant reductions in DFU occurrences between modified and non‐modified insoles, although it was suggested that this result was due to poor patient adherence to the footwear; when non‐adherent patients were removed from the analysis a significant reduction in DFUs was identified.^70^ In some cases, further modifications were needed to preserve offloading efficiency over time, however more research on changes over‐time are needed due to inconclusive results.^72^, ^82^

Continuous offloading is required to combat high reulceration rates and while custom‐made therapeutic footwear, particularly insoles designed using plantar pressure data, have been effective, results between individuals vary.^83^ Further research is needed in order to produce standardised, reliable protocols in design and modification, which can be preserved over time.

Typically, footwear and insoles have been the intervention of choice for reducing high plantar pressures, but a small number of studies providing feedback on high plantar pressures in an attempt to replace what is lost through diabetic peripheral neuropathy offer an alternative intervention (Table. 4).^84^, ^86^ The majority of studies investigating the provision of pressure feedback in individuals with diabetic peripheral neuropathy, do so using visual aids. Few studies detail the methods of providing this feedback, those that do tend to show participants a graph of their average pressure and a highlighted target range usually 40‐80% of baseline.^84^, ^86^ However, in the majority of studies, the pressure data and associated feedback focus on one at‐risk area only, identified as the location of peak pressure while walking. Generally, participants take part in a learning period, which consists of walking followed by the provision of feedback, until a new walking strategy is adopted that offloads the high‐risk area to within the target range. Such studies have reported a significant reduction in pressure applied to the at‐risk area, as a result of a single provision of feedback, and this pressure reduction remained during the follow‐up, the longest retention period assessed being 10 days.^84^, ^86^ However, these studies excluded all foot deformities, whereas York et al.^88^ assessed a higher risk population, excluding only severe foot deformities and reported no lasting significant reductions in plantar pressure. Furthermore, York et al.^88^ provided visual and verbal feedback concerning the forefoot, rather than one at‐risk area. However, a detailed description of the feedback method was not provided and so cannot easily be compared to previous studies. In addition, the effect of the feedback was only assessed over a shorter, one‐week retention period. Nevertheless, such findings suggest participants at higher risk of ulcerating may require more instances of feedback to elicit a positive response.

**Table 4:** Characteristics of studies where plantar pressure feedback is provided

First author (year)	Feedback method	Area where feedback provided	Other areas monitored?	Retention period	Pressure at baseline	Pressure at end of retention	Change to pressure at end of retention	Pressure changes elsewhere	Patient (n =)
aDe Leon Rodriguez (2013)^84^	Graph illustrating plantar pressure target range (40–80% of baseline PPP, for 70% of steps), 1 lab visit	1 at‐risk area	Y	10 days	242 (12)*	167 (11)*	Reduction	Contralateral lateral midfoot increased significantly. The at‐risk lateral midfoot increased slightly	21
bPataky (2000) 85	Audio alarm triggered when pressure exceeded 40% of baseline PPP ‐ worn for 2 weeks	Active ulcer site	N	2 weeks	450	200	Reduction	n/a	1
Pataky (2010)^86^	Graph illustrating plantar pressure target range (40–80% of baseline PPP, for 70% of steps), 1 lab visit	1 at‐risk area	N	10 days	262 (70)	210 (51)	Reduction	n/a	13
cVan (2017)^87^	FEETME pressure map analysis (target pressure 40–80% of baseline for 70% of steps) ‐ 1 visit	1 at‐risk area	Y	6 weeks	—	—	Reduction	No other at‐risk areas developed	6
d ^,^ eYork (2009)^88^	Visual and verbal feedback on gait and forefoot peak pressure, 2 days of feedback	Forefoot	Y	1 week	—	—	No changes	no changes	29
a ^,^ fAbbott (2019)^89^	Continual visual and auditory feedback on sustained high pressure via smartwatch	Both feet (8 sensor sites covering whole foot)	n/a	Continual feedback provided	No pressure data reported, DFU recurrence rates reduced by 71% in the intervention				58

*Note*: Where plantar pressure data provided mean (SD) kPa *(SE). All patients included in the above studies had diabetic peripheral neuropathy.

a Studies monitored pressure across both feet.

b This case‐study provided feedback continuously for 2 weeks to a single participant with an active foot ulcer. The ulcer size reduced from baseline to end of retention.

c Although a reduction in plantar pressure existed at the end of retention, only 50% of steps were below the maximum pressure threshold (80% of baseline), instead of the recommended 70% of steps.

d Plantar pressure at the first MTH significantly reduced 1 day after baseline, however at the end of retention there were no significant changes from receiving feedback.

e This study randomised patients into 2 groups: feedback and no‐feedback. In addition, pressure at 1‐5 MTHs and heel were analysed.

f This study randomised patients into two groups: intervention (receiving continuous pressure feedback) and control (no pressure feedback). Patients in the intervention group received feedback throughout daily‐life when sustained high pressure was detected. No pressure data was reported.

Alternatively, one case study showed promising results for an individual with an active DFU, where feedback provided was in the form of an audio alarm that sounded when pressure exceeded a pre‐determined value.^85^ Following 2 weeks of continuous audio feedback, the participant's DFU size and plantar pressure had reduced, indicating a significant clinical improvement. The results of this single‐participant case study are promising and warrant further investigation through a randomised control trial to validate these positive findings. Although the feedback may be simpler for the participant, this system is again limited to only providing feedback to one area, without the monitoring of overall pressure distribution across the foot. Few studies have addressed this limitation and assess overall pressure distribution in addition to pressure at the specific high‐risk area, in order to identify if any new at‐risk areas develop.^84^, ^87^, ^88^ One study did report a significant increase in pressure to the contralateral lateral mid‐foot following successful off‐loading of the at‐risk area.^84^ Such pressure increase to the contralateral foot may result in the development of a new at‐risk area should the new strategy be continued. However, due to the short follow‐up, as is the case with all previous feedback studies, it is unknown whether such changes to participants' plantar pressure will revert to baseline following a prolonged period. Previous results have shown pressure at the high‐risk area to increase slightly over the retention period, although remaining significantly lower than baseline, perhaps suggesting that a gradual return to baseline may be evident in the absence of sustained feedback.^86^ Such a result also gives reason to provide more regular instances of feedback, rather than providing feedback on a few walking trials, to prevent a return to baseline. Further research is required to investigate long‐term effects of regular feedback on both plantar pressure reduction and associated DFU risk.^90^ With the rise in smart‐technology, we are seeing advancements in pressure‐feedback systems, whereby pressure is analysed and feedback provided continuously.^91^, ^92^ However, such advancements are evident in other treatment areas but until recently were yet to be implemented within diabetes and DFU prevention. A recent prospective, randomised proof‐of‐concept trial saw participants wear an innovative, smart insole system, which provided visual and auditory plantar pressure feedback to the intervention group during daily‐life activities, while a control group had the same sensors without receiving any pressure feedback.^89^ The feedback, which covered eight sensor sites on both feet, was provided via a wrist‐worn smart watch to the intervention group. The smart insole system resulted in a 71% reduction in DFU recurrence in the intervention group and this rose to an 86% reduction in the most highly compliant participants. To the authors' knowledge, this is the first study of its kind to show the effectiveness of a smart insole system designed to measure sustained levels of high, but not peak, plantar pressures and guide regular dynamic offloading in a “real life” situation over a prolonged period for reducing the risk of DFU recurrence.

## 7 CONCLUSIONS AND FUTURE DIRECTIONS

Diabetic foot ulcers are a public health concern, associated with high rates of recurrence and the potential to lead to limb amputation. High plantar pressure is a common risk factor for DFU and patients with a history of DFU are often found to have greater plantar pressures compared to their non‐ulcerated or non‐diabetes counterparts. Vertical plantar pressure is more commonly assessed, however, studies do exist reporting shear pressures, which are of a smaller magnitude and more difficult to assess than the vertical component. At present, shear pressure is often limited to barefoot assessment, whereas vertical plantar pressure has been assessed both barefoot and in‐shoe. While in‐shoe appears to be the most applicable to pressures experienced in daily life limitations still exist. Pressure assessments have been confined to laboratories, with walking being the only weight bearing activity analysed, thus limiting ecological validity. Research into the daily‐life activities of patients with diabetic peripheral neuropathy, although limited, indicates that more time is spent standing and sitting compared to walking. Such findings suggest that perhaps a measure of cumulative pressure over time may be more relevant than the commonly used peak pressure parameter. Custom footwear and insoles are commonly prescribed to offload high plantar pressures; however, further research into the use of pressure to design and modify footwear is required before standardised protocols can be developed. While for the most part, footwear interventions are effective at offloading, results vary between individuals and are only effective when worn regularly. The provision of plantar pressure feedback provides an alternative approach and shows promising results, however, further research is required to understand long‐term effects of feedback, which considers all areas of the diabetic foot. The introduction of smart‐technology, where pressure can be monitored and feedback can be provided on a continual basis, offers a promising method for addressing such shortfalls, with positive results from a randomised proof‐of‐concept trial.

Constraints and other considerations with previous methods of pressure assessment perhaps explain low prediction scores for ulceration. Further pressure analysis, considering both vertical and shear components, outside the laboratory during daily life activities and considering all weight‐bearing activities, is required to improve our understanding of plantar pressures predisposing ulceration. In addition, research is required to investigate whether provision of feedback can result in long‐term beneficial effects, which could ultimately reduce plantar pressure and DFU occurrence.

## CONFLICT OF INTEREST

The authors declare no potential conflict of interest.

## AUTHOR CONTRIBUTIONS

KC planned and wrote the manuscript. NR planned, critically reviewed and edited the manuscript. CA, AB and FB critically reviewed and edited the manuscript. All authors have read and approved the final manuscript.
